# Radiocarbon Releases from the 2011 Fukushima Nuclear Accident

**DOI:** 10.1038/srep36947

**Published:** 2016-11-14

**Authors:** Sheng Xu, Gordon T. Cook, Alan J. Cresswell, Elaine Dunbar, Stewart P. H. T. Freeman, Xiaolin Hou, Piotr Jacobsson, Helen R. Kinch, Philip Naysmith, David C. W. Sanderson, Brian G. Tripney

**Affiliations:** 1 Scottish Universities Environmental Research Centre, East Kilbride, G75 0QF, UK; 2 Institute of Environmental Radioactivity, Fukushima University, Fukushima 960-1296, Japan; 3 Center for Nuclear Technologies, Technical University of Denmark, 4000 Roskilde, Denmark

## Abstract

Radiocarbon activities were measured in annual tree rings for the years 2009 to 2015 from Japanese cedar trees (*Cryptomeria japonica*) collected at six sites ranging from 2.5–38 km northwest and north of the Fukushima Dai-ichi nuclear power plant. The ^14^C specific activity varied from 280.4 Bq kg^−1^ C in 2010 to 226.0 Bq kg^−1^ C in 2015. The elevated ^14^C activities in the 2009 and 2010 rings confirmed ^14^C discharges during routine reactor operations, whereas those activities that were indistinguishable from background in 2012–2015 coincided with the permanent shutdown of the reactors after the accident in 2011. High-resolution ^14^C analysis of the 2011 ring indicated ^14^C releases during the Fukushima accident. The resulted ^14^C activity decreased with increasing distance from the plant. The maximum ^14^C activity released during the period of the accident was measured 42.4 Bq kg^−1^ C above the natural ambient ^14^C background. Our findings indicate that, unlike other Fukushima-derived radionuclides, the ^14^C released during the accident is indistinguishable from ambient background beyond the local environment (~30 km from the plant). Furthermore, the resulting dose to the local population from the excess ^14^C activities is negligible compared to the dose from natural/nuclear weapons sources.

Operation of nuclear reactors for the generation of electrical power can produce various radionuclides by fission within the fuel or by neutron activation in the structural materials and component systems of the reactor. ^14^C is mainly produced in reactors by ^14^N(n, p)^14^C reactions with nitrogen in the fuel, fuel cladding, coolant and moderator as a primary impurity, by ^17^O(n, α)^14^C reactions in oxide fuel, coolant and moderator, and by ^13^C(n, γ)^14^C reactions in graphite moderators. For all types of reactor except pressurised water reactors (PWRs), ^14^C is emitted mainly as CO_2_^1^,^2^. In the case of boiling water reactors (BWRs), as equipped in all units in the Fukushima Dai-ichi nuclear power plant (FDNPP, 37**°**25′23″N, 141**°**01′59″E), 80–95% of the released ^14^C appears to be as CO_2_ and 5–20% as hydrocarbons^2^. Thus, ^14^C emissions from the coolant and moderator are expected during normal operation of a nuclear reactor, whereas accumulated ^14^C in the fuel and fuel cladding is expected to be emitted at the time of melt-down of the reactor. Thus, it is highly likely that ^14^C emission at the time of the accident might become much higher than during normal operations. Elevated ^14^C activities have been reported around many nuclear reactor sites worldwide during normal operations^3^. As an example of a nuclear accident, the Chernobyl NPP accident resulted in a maximum ^14^C excess of 281.6 Bq kg^−1^ C within the local environment, while the affected area, with a ^14^C excess of >10 Bq kg^−1^ C, extended to >40 km from the plant^4^,^5^. Accordingly, the release of ^14^C from the Chernobyl accident was estimated to be 44 PBq^5^. The large release of ^14^C from the Chernobyl accident is mainly attributed to the high production of ^14^C in the graphite moderators through the ^13^C(n, γ)^14^C reaction and the burning of the graphite during the accident.

Since the FDNPP accident on 11^th^ March 2011 (Fig. 1), numerous studies of Fukushima-derived radionuclides in the worldwide environment have been conducted to reconstruct the history, levels and geochemical behaviours of the releases^6^,^7^. However, these studies mainly focused on relatively short-lived radionuclides such as ^134,137^Cs and ^131^I. Measurements of long-lived radionuclides such as ^14^C, ^36^Cl, ^129^I or ^236^U are still relatively limited and in particular, ^14^C remains as one of Fukushima’s most understudied radionuclides^7^. These “forgotten” radionuclides are mainly of academic interest because of their applicability as environmental tracers. However, ^14^C is one of the most important radionuclides for regional radiological assessment because it gives a significant fraction of the effective dose to the general public through the atmosphere-agricultural food-ingestion pathway.

Although ^14^C is not expected to constitute one of the most hazardous emissions from the Fukushima accident, from scientific and social points of view it is necessary to assess if there were any ^14^C releases during the accident. If ^14^C was indeed released, questions arise of how much was released and how it was distributed. However, such investigations have been delayed because there are not many suitable environmental materials that can capture the gaseous ^14^C release.

In our previous work on ^14^C analysis of tree rings from a 30-year-old Japanese cedar from Iwaki, approximately 50 km southwest of the FDNPP^8^, a local fossil fuel effect on the ^14^C activity was observed and identified as originating from increasing traffic on two nearby expressways that were opened in the 1990 s. In addition, a small but visible ^14^C increase observed in the 2011 tree ring was postulated to be caused by the release from the Fukushima accident. However, our later study on a 50-year-old Japanese cedar from Okuma, ~1 km southwest of the FDNPP confirmed significant ^14^C discharges during normal operations of the FDNPP between 1976 and 2010, but no visible ^14^C excess in 2011, at least in the southwest direction^9^. Recently, Povinec *et al*.^10^ discussed possible ^14^C contributions of Fukushima origin to the activity in offshore seawater collected on 3–7 June 2011. The profiles at two stations (37°30′N, 144°00′E and 38°00′N, 143°00′E) showed ∆^14^C values of around −20‰ at 100–200 m depth, implying approximately 9% contributions of Fukushima origin, on the assumption of a ∆^14^C value of −115‰ at 400–500 m depth as the ambient background value. Thus, the question remains unresolved as to whether the Fukushima accident in 2011 released significant amounts of ^14^C into the environment. If ^14^C was indeed released during the accident, the absence of a ^14^C signal in the Okuma 2011 tree ring sample might be due to the limited amount of ^14^C released from the damaged reactors during the accident, and/or the prevailing wind direction being away from Okuma during the releases, and/or the timing of the release being too early in relation to the main onset of photosynthetic activity. Within the context of this background, the current work (1) investigates ^14^C levels before and after the accident to identify any release from the accident by measuring ^14^C in annual tree rings of Japanese cedar trees from sites 2.5–38 km in a north and northwest direction from the FDNPP, (2) compares these values with the global and local atmospheric ^14^CO_2_ datasets, and (3) briefly discusses the difference in the magnitude of ^14^C released during the Fukushima accident compared to that at the Chernobyl NPP in 1986.

To achieve these objectives, it is essential to understand pre-Fukushima atmospheric ^14^C levels observed in Japan so as to compare them with our tree ring data. In this study, the global ^14^C concentrations, which are represented by the Schauinsland Station (SIL), an atmospheric CO_2_ monitoring station in Germany^11^, are considered to be the ambient background values. This justification is based on the good agreement between the long-term global atmospheric data and regional atmospheric data in Japan, including CO_2_, tree ring and rice grain samples measured throughout Japan. Detailed discussion of this was made in our previous study^9^.

In summary, the analytical results of this study reveal that ^14^C, with a maximum specific activity of 42.4 Bq kg^−1^ C above the natural background, was released during the Fukushima accident and that the activity decreased with increasing distance from the plant. Unlike other Fukushima-derived radionuclides, the released ^14^C is limited to the local environment (probably approximately 30 km northwest from the FDNPP). The resulting dose to the local population is negligible compared to that from natural/nuclear weapons sources.

## Results

**Figure 1:**
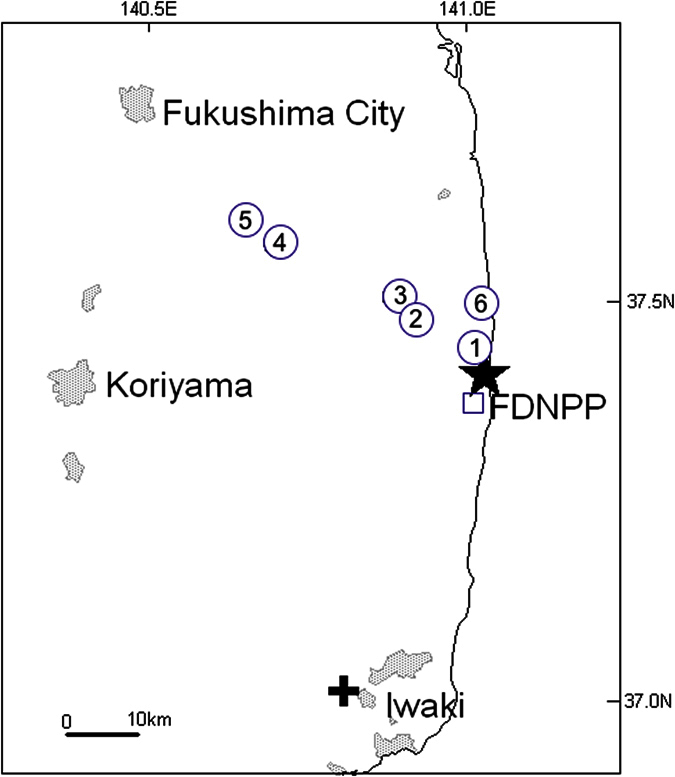
Map showing FDNPP and sampling sites in this study marked with open circles and numbers corresponding those in Table 1. The Iwaki and Okuma tree rings in our previous studies are also shown with cross and open square, respectively^8^,^9^. The map was produced using software MapInfo Professional v5.5 using data from Digital Chart of the World (http://worldmap.harvard.edu/data/geonode:Digital_Chart_of_the_World).

Figure 1 shows the sampling locations in this study, in addition to other sites used in previous studies. The analytical results (δ^13^C and ^14^C) are listed in Table 1. The ^14^C data are expressed as fraction modern (F^14^C) where modern is 0.7459 times the activity of the primary standard sample, NBS oxalic acid II (SRM-4990C). The corresponding specific activities of ^14^C are also given in Table 1. The weighted mean F^14^C of the eleven humic acid secondary standards (as used in the Fifth International Radiocarbon Inter-comparison (VIRI) as Sample T)^12^, employed to monitor accuracy and precision, is 0.6588, with a relative standard deviation of 0.2%. This is in excellent agreement with the consensus value of 0.6587 ± 0.0035 and accordingly confirms the overall ~0.2% precision and accuracy of the measurements in this study.

**Figure 2:**
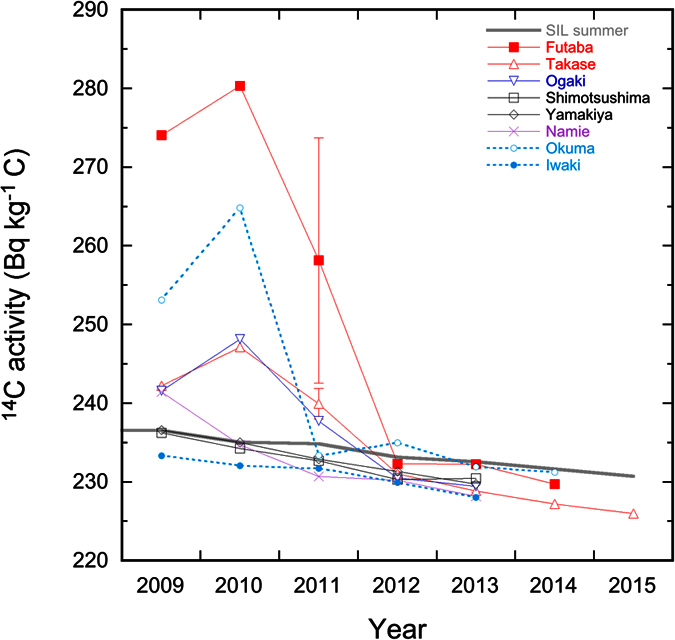
Temporal variations of ^14^C in tree-ring and atmospheric CO_2_. The data points of the global atmospheric ^14^C values are the average value of May-August of the SIL^11^. Note that the SIL data points of 2013–2015 are extrapolated from the exponential fitting of ^14^C data in period 2000–2012. The data point of year 2011 ring in Futaba is shown as the average value with 1σ standard deviation of the six sub-rings, whereas that in Takase is the average value of the early and late-wood. The 1σ uncertainties of other samples are within the symbol point. The blue and dotted lines stand for the tree rings from Iwaki^8^ and Okuma^9^.

Significant variations in the ^14^C specific activities of the tree rings were observed, ranging from 280.4 ± 0.6 Bq kg^−1^ C in the 2010 ring from Futaba to 226.0 ± 0.5 Bq kg^−1^ C in the 2015 ring from Takase. Figure 2 shows the temporal variations in ^14^C specific activity in tree rings from the six sites in this study, together with global atmospheric ^14^CO_2_ values from SIL, and ^14^C measurements from local tree rings in Iwaki and Okuma^8^,^9^. The excess ^14^C activity in this study can be calculated by subtracting the ambient global atmospheric ^14^C values at SIL, with any resulting negative values most likely caused by a local fossil fuel effect on the ^14^C activity (Table 1).

One of the most distinctive features in the present dataset is that all the rings, except one, within the post-accident 2012–2015 period, are distributed along the SIL curve or between the SIL and Iwaki curves. The former pattern suggests that the local atmospheric ^14^C record follows the exponential decline in global atmospheric ^14^CO_2_, whereas the latter reveals that the atmospheric ^14^C in the studied area might have been slightly affected by additional local fossil fuel combustion, as demonstrated in Iwaki^8^. In comparison with the global atmospheric ^14^C levels, the ^14^C depletion observed in Iwaki has been attributed to the two nearby expressways, open since the 1990s^8^. This may not be the case for the samples from Takase and Ogaki (both of which are close to the Joban expressway), because part of the expressway construction, between Joban-Tomioka IC (just south of FDNPP) and Yamamoto IC (north of Soma city), was not completed at the time of the Fukushima nuclear accident in 2011. This part was finally opened in March 2015. In addition, the southern part between Hirono IC and Joban-Tomioka IC was temporarily closed until radionuclide decontamination finished in 2014. Therefore, like other sites worldwide, the cause of the ^14^C depletion at Takase and Ogaki could be attributed to CO_2_ emissions from fossil fuel combustion derived from local industry, traffic and household heating, etc. In comparison with the SIL curve, the fact that no significantly elevated ^14^C pulse was observed in post-accident rings is in good agreement with the shutdown of all reactors in the FDNPP after the 2011 accident. In contrast, the ^14^C concentrations in pre-accident 2009–2010 rings show complex features. The ^14^C concentrations in the 2010 ring from Namie and the 2009–2010 rings from Shimotsushima and Yamakiya are consistent with the SIL values, again indicating the exponential decline in the global atmospheric ^14^CO_2_ activity. On the other hand, like the enhancement in ^14^C prior to the accident, observed in Okuma tree rings^9^, the elevated ^14^C in the 2009 rings from Futaba, Takase, Ogaki and Namie, and in the 2010 rings from Futaba, Takase and Ogaki, confirm the existence of ^14^C discharges during routine reactor operations, which is consistent with observations at other nuclear sites using similar technologies^3^. It should be pointed out that the ^14^C activities in 2009 and 2010 in the Futaba sample are higher than those observed in the Okuma sample by 8% and 6%, respectively. This difference can be attributed to the sample location. Okuma is located in the crosswind direction of the FDNPP while Futaba is located along the prevailing downwind direction. This observation confirms that meteorological conditions play an important role in ^14^C dispersion, as observed at other nuclear sites worldwide^3^.

**Figure 3:**
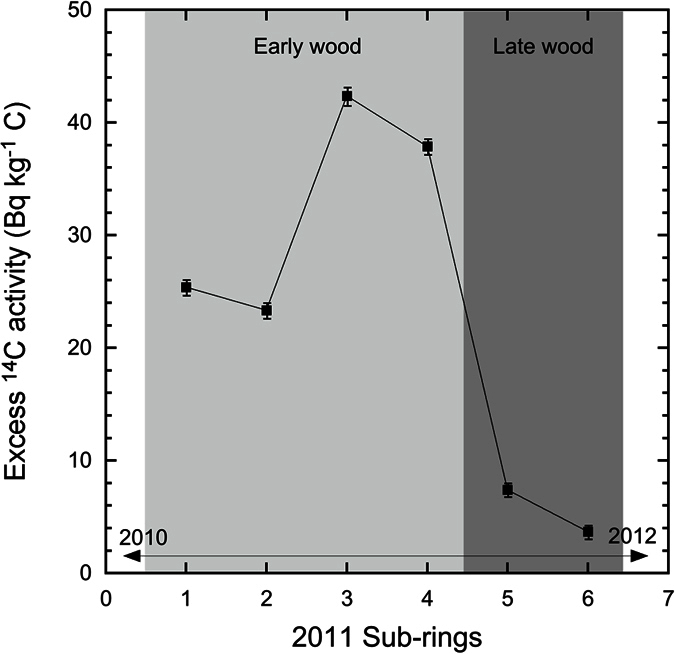
Variations of the observed excess ^14^C activity in the sub-sections of the 2011 ring from Futaba sample.

Another distinctive feature in the present dataset can be observed in the 2011 rings. Unlike the ^14^C specific activities from the 2011 tree rings from Shimotsushima, Yamakiya, Namie, Okuma and Iwaki, which are indistinguishable from the global atmosphere (Fig. 2), samples from Futaba, Takase and Ogaki have significantly higher ^14^C specific activities than the corresponding SIL values, with excess ^14^C activities varying from 3.0 ± 0.7 Bq kg^−1^ C in Ogaki to 42.4 ± 0.8 Bq kg^−1^ C in Futaba (Sub-section 3) (Table 1). The six sub-sections of the 2011 ring at Futaba show large ^14^C variations (Table 1 and Fig. 3). The excess ^14^C activity sharply increased from 25.4 ± 0.7 and 23.3 ± 0.7 Bq kg^−1^ C in the oldest two sub-sections to 42.4 ± 0.8 and 37.9 ± 0.7 Bq kg^−1^ C in the two middle sections, and then immediately decreased to 7.4 ± 0.6 and 3.7 ± 0.6 Bq kg^−1^ C in the most recent parts of the ring. Although it is not possible to identify the exact growing periods for each sub-section, as described above, the ring profile of the Futaba sample shows that the first four and later two sub-sections belong to the early wood and late wood, respectively. This means that the excess ^14^C activity in the early wood (23–42 Bq kg^−1^ C) is 3–10 times higher than the late wood (4–7 Bq kg^−1^ C). Similarly, the ^14^C concentration in the early wood from Takase is about 2 times higher than that in the late wood. Such a pattern has been observed in the 1986 rings collected from three sites within 2–12 km of the Chernobyl nuclear power plant, as a result of the accident that occurred on the 26^th^ April 1986^4^.

## Discussion

### ^14^C releases during the Fukushima accident

The elevated ^14^C activities in the 2009 and 2010 rings from Futaba, Ogaki and Takase confirm the discharge of ^14^C during routine reactor operations of the FDNPP, as observed in the Okuma tree rings^9^. On the other hand, the ^14^C levels in the post-accident 2012–2015 rings are indistinguishable from the general atmospheric values and this strongly suggests a fast response to the cessation in ^14^C discharges due to the shutdown of the FDNPP after the 2011 accident. However, the cause of the elevated ^14^C in the 2011 rings most probably differs from that of the pre-accident. There are several possibilities that could result in the elevated ^14^C activities in the 2011 rings around Fukushima: (1) ^14^C translocation or stored photosynthate from the previous year, (2) ^14^C discharges from normal operations prior to 11^th^ March 2011, and (3) ^14^C release from the Fukushima accident.

The use of stored photosynthate from the previous year (autumn and winter) has been studied previously in Sitka spruce and found to be relatively unimportant^13^. In addition, our previous study indicated elevated ^14^C activities in the Okuma tree rings from 1976 to 2010^9^. However, the ^14^C activity abruptly decreased from 264.8 Bq kg^−1^ C (an excess of approx. 39.4 Bq kg^−1^ C) in the 2010 ring to a background level of 233.4 Bq kg^−1^ C in the 2011 ring. This fact indicates an immediate response to the shutdown of the FDNPP after the accident. Moreover, the ^14^C activities in the 2012 rings from Futaba, Takase and Ogaki declined abruptly from high values in the 2011 rings to a background value. These observations support a negligible stored photosynthate contribution to the 2011 ring in Okuma, and the 2012 rings in Futaba, Takase and Ogaki, from the previous years, which had significantly higher ^14^C activities.

^14^C produced in the coolant and moderator may be discharged into the immediate environment during normal operations, as demonstrated in the 2009 and 2010 rings within this study and the 1976–2010 rings at Okuma^9^. There is a positive correlation between the excess ^14^C activity and the average annual energy output from the six BWRs during the period of full operations within the FDNPP^9^:


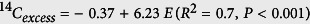


where ^*14*^*C*_*excess*_ and *E* are the excess ^14^C activity in the tree ring (Bq kg^−1^C) and the annual electrical energy output (GWe), respectively. Based on the annual electrical energy output (*E* = 0.35 GWe) generated during the period between 1^st^ January and 11^th^ March 2011^14^, the excess ^14^C activity in the 2011 ring can be estimated to be approximately 2 Bq kg^−1^ C from the six BWRs. Obviously, this value is more than one order of magnitude lower than the actual maximum ^14^C excess (42.4 ± 0.8 Bq kg^−1^ C) measured in the third sub-section of the 2011 ring. Therefore, the estimation suggests that less than 5% of the excess ^14^C in the 2011 ring was from discharges during the routine operation of the FDNPP prior to the accident. This conclusion is further supported by the seasonal variations in wind direction. Figure S1 shows 72 h (3 day) air mass forward trajectory analysis for starting altitudes of 0 m above ground level (AGL) during the period between 1^st^ January and 11^th^ March 2011, calculated from the FNL database of the National Ocean and Atmospheric Administration (NOAA) and simulated by using the Hybrid Single-Particle Lagrangian Integrated Trajectory (HY-SPLIT) model^15^,^16^. These data suggest that in the costal area of Fukushima Prefecture, the wind direction was prevailing to the east or southeast during winter and to north or northwest during summer. Thus, such meteorological conditions would limit the capture of the discharged ^14^C along the northwest direction during the winter season in 2011 (January to early March). Also, as the majority of ^14^C in an annual tree ring is generally considered to be captured by photosynthesis during the summer season, ^14^C discharged from the FDNPP between January and March 2011 would be subject to much less captured due to the lower rate of photosynthesis during this period.

Thus, ^14^C release during the accident should be considered as the main source of the excess ^14^C activity in the 2011 tree ring. As described above, ^14^C can be produced within nuclear reactors by neutron reactions with nitrogen, oxygen and carbon in fuel, fuel cladding, and coolant and moderator. A summary of typical ^14^C production rates in various types of reactor has been given by IAEA^2^. For the BWR-type reactor, like those employed at the FDNPP, the calculated ^14^C production rates are 470, 630 and 190 GBq·GW(e)^−1^ a^−1^ in fuel, fuel cladding and coolant and moderator, respectively^2^. Therefore, the ^14^C produced in the damaged reactors (Units 1–3 in FDNPP) can be estimated if the electrical energy output and actual fuel-burn time in each reactor can be obtained.

As discussed in Miyake *et al*.^17^, the reactors in the FDNPP were normally shut down for regular inspection after every 300–400 days of operation. About 20% of the used fuel assemblies are replaced with new assemblies during each regular inspection. From the history of operation times and the number of replaced fuel assemblies, the actual fuel-burn time at the time of the Fukushima accident for Units 1–3 was estimated to fall between 850 and 1000 days^17^. Thus, based on the reported electrical energy output^14^, the calculated ^14^C production during the period of 850–1000 days before the accident would be 450–700, 600–940 and 180–280 GBq in fuel, fuel cladding and coolant and moderator, respectively. This reveals that a total ^14^C activity of 1.0–1.6 TBq might have accumulated in fuel and fuel claddings within Units 1–3 of the FDNPP.

The design of BWRs leads to a continuous release of ^14^C mainly produced in the coolant and moderator through the discharge stack during normal reactor operations. The ^14^C produced in the solid materials (i.e. fuel and fuel cladding) is generally not released during normal operation, however, the earthquake and subsequent tsunami that occurred on the 11^th^ March 2011 damaged reactor Units 1–3 and in turn resulted in release of the ^14^C stored within these reactor units.

Most of the gaseous releases of ^14^C, in the form of ^14^CO_2_ can be assimilated by plants through photosynthesis. It is well known that the start of net photosynthesis in spring mainly depends on the ambient temperature. Positive net photosynthesis will occur as long as the water in the soil is no longer frozen^18^. The majority of the atmospheric releases took place between the 12^th^ and 22^nd^ March, with a maximum release phase from the 14^th^ to 17^th^ March. The local average atmospheric temperature between 08:00 and 18:00 hours, during the period of 11^th^–25^th^ March 2011, was about 5 °C (http://www.data.jma.go.jp). Thus, local weather conditions would enable the released ^14^CO_2_ to be captured by photosynthesis although the rate might be low. This is supported by spatial and temporal investigations which demonstrated that the light-saturated photosynthetic capacity of *Cryptomeria japonica* fell to only half of its maximum value in winter^19^.

In comparing ^14^C concentrations in tree rings with those of atmospheric CO_2_, it is important to understand at what time the cellulose in a certain part of a tree ring was synthesized from the CO_2_ in the air. It has been reported that the early wood of each annual ring of a Kiso Hinoki tree (*Chamaecyparis obtusa, Japanese cypress*), grown in a rural forest at Gifu, Japan, was formed from the beginning of May to the end of July by assuming a uniform growth rate^20^. On the basis of consistent ^14^C data between the tree ring and global atmospheric CO_2_, it was explained that the time-lag between the fixation of CO_2_ by the tree through photosynthesis and cellulose formation using the photosynthetic products seems to be negligible during the growing season of the tree. Similar results for an oak tree grown in a suburb of Uppsala in Sweden showed that the total growing time was normally from June to the end of August, and that there was no clear evidence for any delay in ^14^C activity between the atmospheric CO_2_ and the cellulose fraction in the tree, on a time scale of weeks^21^. However, it has also been demonstrated that ^14^C values for tree ring cellulose in Sitka spruce from the United States Pacific Coast seemed to follow those of atmospheric CO_2_ with a delay of 5 to 6 weeks^13^. The Fukushima accident occurred on 11^th^ March 2011 and the radioactive releases most likely lasted until the end of the month. If the early wood was formed from the beginning of March to the end of July by assuming an equal growth rate, and if the maximum excess in ^14^C corresponds to the maximum radioactive release period, the third and fourth sub-sections of the 2011 ring of the Futaba sample, which contained the highest excess ^14^C activities, would be formed between May and June 2011. If this is the case, our results seem to be compatible with a delay of around 5–6 weeks.

### Spatial distribution of the Fukushima-derived ^14^C

**Figure 4:**
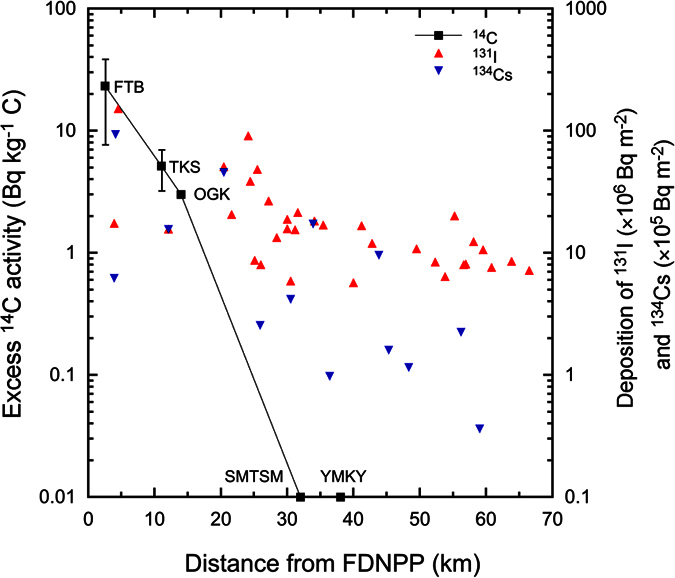
Spatial distribution of the excess ^14^C activity in the 2011 rings and selected short-lived radioactive isotopes (^131^I and ^134^Cs) in soil^22^,^23^ to the northwest of the FDNPP. Note that the average values with 1σ uncertainty of sub-samples from Futaba and Takase are shown. FTB: Futaba, TKS: Takase, OGK: Ogaki, SMTSM: Shimotsushima, and YMKY: Yamakiya.

There was a spatial trend in the ^14^C released during the Fukushima accident. Two samples collected southwest of the FDNPP (Okuma and Iwaki) and one sample north of FDNPP (Namie) showed no visible ^14^C excess in the 2011 ring (Fig. 2). However, an excess in ^14^C was clearly observed along the northwest direction from the FDNPP, gradually decreasing from an average of 23.4 Bq kg^−1^ C near the FDNPP (Futaba) to 0 Bq kg^−1^ C around 30 km from the plant (Fig. 4). Such a ^14^C distribution pattern is locally consistent with other fission-induced radionuclides such as ^131^I and ^134,137^Cs^22^,^23^,^24^. This feature confirms that the wind direction is a major factor in controlling the ^14^C distribution. This is particularly true for the Okuma sample that is located only around 1 km from the FDNPP, but in a cross-wind direction. In the early stages of the Fukushima accident, the prevailing wind direction was from the west and in the later stages from the southeast (Supplementary Table S1 and Fig. S2). Based on these meteorological conditions, it can be concluded that the majority of the released ^14^C was dispersed towards the Pacific Ocean and the remainder was distributed inland along a northwest direction. More detailed analysis of the excess ^14^C spatial distribution by the best fitting exponential plot of the data shows that influence of the Fukushima-derived ^14^C is probably limited to within less than 30 km from FDNPP (Fig. 4). A similar spatial pattern has been reported at other nuclear sites including Chernobyl^5^,^25^. At these sites, the distances influenced by nuclear-derived ^14^C were usually limited to tens of kilometres.

In estimating radioactive exposure at the time of the accident, exposure by inhalation of gaseous ^131^I is rather difficult to estimate due to a lack of direct information on levels and transport. Considering the fact that gaseous CO_2_ may behave closer to gaseous iodine than to particulate forms of radioactive discharges, the distribution of ^14^C might provide us with information for estimating gaseous iodine exposure. Indeed, the spatial distribution of ^14^C has some similarity to that of ^134,137^Cs and ^131^I but only in that there was a decrease in activity with increasing distance from the plant (Fig. 4). However, this figure also demonstrates that while the ^14^C activity falls off to a value indistinguishable from background by around 30 km, the ^134^Cs and ^131^I activities are measureable at >60 km. The spatial distributions of the latter radionuclides in the terrestrial environment were thought to be influenced not only by wind transport but also by precipitation in each location as well as their ratios as particulate forms in the air^6^. In contrast, the ^14^C is fixed by photosynthesis and cellulose formation, and expected to remain in the same locations, whereas the post-depositional behaviours of ^134,137^Cs and ^129,131^I are prone to redistribution. Therefore the relationships between these radionuclides might be more complex. It should also be noted that whereas there will be no background level of ^131^I or ^134^Cs, the ^14^C is being measured against an existing and significant global background level of activity and this could be a contributing factor in determining an excess activity. Nevertheless, to clarify these findings, more detailed analysis should be conducted in future.

In addition, the spatial distribution of ^14^C in tree rings might also provide us with a rough estimate of the possible flow of emitted radioactivity at the time of the accident. In particular, investigation of the distribution of ^14^C around current nuclear power plants worldwide might help us to estimate risks and to design efficient measures for radiation protection in the case of accidents.

### Comparison of the Fukushima- and Chernobyl-derived ^14^C

It would be interesting to estimate the total ^14^C activity released into the atmosphere during the Fukushima accident and to compare it with the Chernobyl release. However, any detailed discussion of this is beyond the limited data available within this study. More spatial sampling and a model calculation are required to estimate the total releases of ^14^C and will form the basis of a future study. Nevertheless, a rough comparison of ^14^C releases between the accidents from Fukushima and Chernobyl can be made as follows.

The Fukushima accident resulted in the release of ^14^C, with excess values up to 42.4 Bq kg^−1^ C at ~3 km from the damaged reactors and with a significantly affected (^14^C_excess_ > 5 Bq kg^−1^ C) area up to ~15 km to the northwest. However, the Chernobyl NPP accident resulted in a maximum ^14^C excess of 281.6 Bq kg^−1^ C within the local environment, while the affected area, with a ^14^C excess of >10 Bq kg^−1^ C, extended to >40 km from the plant^4^,^5^. Therefore, in comparison with the Chernobyl accident, releasing a ^14^C activity of 44 PBq^5^, the observed Fukushima-derived ^14^C release was very likely significantly smaller.

This difference can be reasonably explained by the different type of reactors employed at the Chernobyl and Fukushima NPPs, although the scale of the accidents was officially the same. The Chernobyl NPP was equipped with a graphite moderator which could produce ^14^C at a rate of >3000 GBq·GW(e)^−1^·a^−1^ alone^2^. However, all three damaged reactors in the FDNPP are of the BWR-type which have a total ^14^C production rate of 1300 GBq·GW(e)^−1^·a^−1 ^2. Thus, it is clear that the lower ^14^C production rate in the FDNPP would result in a much lower release of ^14^C compared to Chernobyl where, in particular, burning of graphite significantly enhanced the ^14^C releases.

### Implication of dose contribution to the public from the Fukushima-derived ^14^C

The effective dose to individuals due to ^14^C releases can be estimated following the model proposed by the IAEA^26^. In this model, it is assumed that the main ^14^C exposure pathway for individuals is via ingestion of foodstuffs (atmospheric carbon assimilation by photosynthesis, leading to its presence in the food chain), and that ^14^C activity equilibrium has been reached between atmospheric ^14^CO_2_ and foodstuffs. The effective dose is therefore proportional to the specific activity of the foodstuffs. However, such estimation is also beyond the scope of this study because information such as the fraction of the food carbon coming from local production and the dose coefficient are lacking. Nevertheless, a qualitative assessment of the dose contribution from Fukushima-derived ^14^C can be determined due to the fact that the activity level of the Fukushima-derived ^14^C is significantly lower than that produced by the global weapons tests in the 1950 s and 1960 s. This finding indicates that, unlike the large releases of other radionuclides (*i.e.*, radiocesium and radioiodine) during the accident^22^,^23^,^27^, the influence of ^14^C was not only limited to the local environment, probably within ~30 km from the FDNPP, but also, the resulting dose to the population is negligible compared to the natural/nuclear weapons dose.

## Methods

### Sampling site and materials

Five blocks (sample Nos 1 and 3–6) of Japanese cedar (*Cryptomeria japonica*), cut from large branches of living cedar trees, were collected from Futaba, Ogaki, Shimotsushima, Yamakiya and Namie on the 11^th^–13^th^ November 2014 (Fig. 1). In addition, using a 10 mm diameter tree-ring borer, one core sample (sample No. 2) was collected from a cedar tree in Takase on the 18^th^ May 2015. These cedar trees are 10–30 years old approximately and >3 m in height. Care was taken to sample about 2 m above ground level to avoid any possible local canopy effects from ^14^C released from soils^28^. Being within 40 km from the FDNPP, the sampling sites were heavily contaminated by radionuclides released during the 2011 Fukushima accident^22^,^23^,^24^.

Short-term wind direction data (every 10 minutes) were obtained from several meteorological stations near the FDNPP. From these data, the frequency/direction of the wind was determined for the total cedar tree growth period. The photosynthetic period was estimated to be between the daylight hours of 08:00–18:00 (JST) approx. Due to a power failure in the FDNPP and its adjacent areas during the accident, the available meteorological data could only be obtained intermittently from the stations at Namie (10 km northwest of FDNPP), Iitate (38 km northwest of FDNPP) and Kawauchi (20 km southwest of FDNPP), the nearest available stations to the FDNPP. The main wind directions recorded in these stations are summarized in Supplementary Table S1 and two rose diagrams of the wind directions during the period of the two radioactive plumes (14^th^–15^th^ and 21^st^–22^nd^ March 2011) are shown in Supplementary Fig. S2.

### Chemical preparations and ^14^C measurements

In the case of the Futaba sample (No. 1), individual annual rings were cut for the years 2009–2010 and 2012–2014. To investigate any ^14^C seasonal/temporal variations, six approximately equal sub-sections of the 2011 ring were separated. Due to the higher growth rate in the early wood compared with the late wood, sub-sections 1–4 fell within the early wood period, while sub-sections 5–6 are the late wood. In the case of the sample from Takase (No. 2), the early- and late-wood in the 2011 ring were cut separately, in addition to entire annual rings for years 2009–2010 and 2012–2015. The entire annual rings representing years 2009–2013 were cut for the samples (Nos 3–6) from Ogaki, Shimotsushima, Yamakiya and Namie.

The alpha cellulose fraction of each tree ring was chemically extracted and prepared for ^14^C analysis. Detailed procedures for the chemical extraction, combustion, CO_2_ purification and graphite preparation are described elsewhere^29^; however, a brief description is as follows: The alpha cellulose fraction was isolated using a combination of solvent extraction, bleaching with hypochlorite solution and acid-base-acid extraction. The isolated alpha cellulose was then combusted at 850 °C in an evacuated quartz tube containing CuO (as the source of oxygen) and Ag foil to mop up halides and other contaminants. The resulting CO_2_ was cryogenically purified and a 3 ml sub-sample converted to graphite for subsequent ^14^C measurement by accelerator mass spectrometry (AMS). A further aliquot of CO_2_ from each sample was measured for δ^13^C by conventional isotope ratio mass spectrometry (IRMS) using a VG SIRA 11^29^. In addition to the primary standard (SRM-4990C), in-house secondary standard samples comprising bulk barley mash from the Third International Radiocarbon Inter-comparison (TIRI Sample A with a fraction modern value of 1.1635)^30^, and individually combusted humic acid standards (VIRI Sample T with a fraction modern value of 0.6587 ± 0.0035)^12^, were used for quality control. Individually combusted interglacial wood samples (Heidelberg wood, VIRI Sample K) acted as the background standard sample. These standards were prepared and measured by the same procedures as the tree ring samples in order to assess the total uncertainty and background in the ^14^C measurements.

^14^C activities were calculated by ^14^C/^13^C ratio measurements using a National Electrostatics Corporation 5MV Tandem AMS in the SUERC AMS Laboratory, a Natural Environmental Research Council (NERC) Recognised Facility^31^. All but the background wood samples were measured to achieve better than 0.2% uncertainties for both the ^14^C counting statistics and the repeat measurement scatter.

## Additional Information

**How to cite this article**: Xu, S. *et al*. Radiocarbon Releases from the 2011 Fukushima Nuclear Accident. *Sci. Rep.* **6**, 36947; doi: 10.1038/srep36947 (2016).

**Publisher’s note**: Springer Nature remains neutral with regard to jurisdictional claims in published maps and institutional affiliations.

## Supplementary Material

Supplementary Information

## Figures and Tables

**Table 1:** Analytical results of tree rings from Fukushima, Japan*.

No.	Lab code (SUERC-)	Year	Wood fraction	δ^13^C (‰)	^14^C Fraction modern	^14^C Fraction absolute modern	^14^C activity (Bq kg^−1^ C)	^14^C background** (Bq kg^−1^ C)	Excess ^14^C activity (Bq kg^−1^ C)
1. Futaba (37°26.416′N, 141°1.077′E, 2.5 km NW of FDNPP)									
	59934	2014	Whole ring	−27.2	1.0245 ± 0.0019	1.0166 ± 0.0019	229.8 ± 0.4	231.1 ± 0.7	−1.3 ± 0.8
	59935	2013	Whole ring	−27.2	1.0357 ± 0.0019	1.0278 ± 0.0019	232.3 ± 0.4	232.1 ± 0.7	0.2 ± 0.8
	59936	2012	Whole ring	−27.7	1.0358 ± 0.0021	1.0280 ± 0.0021	232.3 ± 0.5	233.1 ± 0.4	−0.8 ± 0.6
	59937	2011	Sub-ring (6)	−27.3	1.0631 ± 0.0018	1.0553 ± 0.0018	238.5 ± 0.4	234.8 ± 0.5	3.7 ± 0.6
	59938	2011	Sub-ring (5)	−27.6	1.0797 ± 0.0020	1.0718 ± 0.0020	242.2 ± 0.4	234.8 ± 0.5	7.4 ± 0.6
	59944	2011	Sub-ring (4)	−27.7	1.2156 ± 0.0021	1.2066 ± 0.0021	272.7 ± 0.5	234.8 ± 0.5	37.9 ± 0.7
	59945	2011	Sub-ring (3)	−27.5	1.2355 ± 0.0026	1.2264 ± 0.0026	277.2 ± 0.6	234.8 ± 0.5	42.4 ± 0.8
	59946	2011	Sub-ring (2)	−26.0	1.1507 ± 0.0023	1.1422 ± 0.0023	258.1 ± 0.5	234.8 ± 0.5	23.3 ± 0.7
	59947	2011	Sub-ring (1)	−26.6	1.1599 ± 0.0023	1.1513 ± 0.0023	260.2 ± 0.5	234.8 ± 0.5	25.4 ± 0.7
	59948	2010	Whole ring	−27.2	1.2495 ± 0.0025	1.2405 ± 0.0025	280.4 ± 0.6	235.0 ± 0.6	45.3 ± 0.8
	59954	2009	Whole ring	−27.1	1.2214 ± 0.0024	1.2127 ± 0.0024	274.1 ± 0.6	236.6 ± 0.5	37.5 ± 0.8
2. Takase (37°28.0862′N, 141°55.4637′E, 11 km NW of FDNPP)									
	63147	2015	Whole ring	−23.9	1.0078 ± 0.0022	0.9999 ± 0.0022	226.0 ± 0.5	230.2 ± 0.8	−4.2 ± 0.9
	63153	2014	Whole ring	−23.6	1.0132 ± 0.0022	1.0054 ± 0.0022	227.2 ± 0.5	231.1 ± 0.7	−3.9 ± 0.9
	63154	2013	Whole ring	−24.6	1.0204 ± 0.0022	1.0127 ± 0.0022	228.8 ± 0.5	232.1 ± 0.7	−3.3 ± 0.8
	63155	2012	Whole ring	−24.9	1.0300 ± 0.0022	1.0223 ± 0.0022	231.0 ± 0.5	233.1 ± 0.4	−2.1 ± 0.6
	63156	2011	Late ring	−25.1	1.0634 ± 0.0023	1.0556 ± 0.0023	238.6 ± 0.5	234.8 ± 0.5	3.8 ± 0.7
	63157	2011	Early ring	−23.8	1.0757 ± 0.0023	1.0678 ± 0.0023	241.3 ± 0.5	234.8 ± 0.5	6.5 ± 0.7
	63163	2010	Whole ring	−26.0	1.1014 ± 0.0020	1.0934 ± 0.0020	247.1 ± 0.4	235.0 ± 0.6	12.1 ± 0.7
	63164	2009	Whole ring	−24.5	1.0794 ± 0.0023	1.0717 ± 0.0023	242.2 ± 0.5	236.6 ± 0.5	5.6 ± 0.7
3. Ogaki (37°30.391′N, 140°55.021′E, 14 km NW of FDNPP)									
	59965	2013	Whole ring	−24.4	1.0230 ± 0.0019	1.0153 ± 0.0019	229.4 ± 0.4	232.1 ± 0.7	−2.7 ± 0.8
	59966	2012	Whole ring	−24.9	1.0275 ± 0.0018	1.0199 ± 0.0018	230.5 ± 0.4	233.1 ± 0.4	−2.6 ± 0.6
	59967	2011	Whole ring	−25.1	1.0598 ± 0.0021	1.0520 ± 0.0021	237.8 ± 0.5	234.8 ± 0.5	3.0 ± 0.7
	59968	2010	Whole ring	−25.2	1.1061 ± 0.0022	1.0981 ± 0.0022	248.2 ± 0.5	235.0 ± 0.6	13.2 ± 0.8
	59974	2009	Whole ring	−24.9	1.0767 ± 0.0019	1.0690 ± 0.0019	241.6 ± 0.4	236.6 ± 0.5	5.0 ± 0.6
4. Shimotsushima (37°34.415′N, 140°43.743′E, 32 km NW of FDNPP)									
	59975	2013	Whole ring	−26.0	1.0274 ± 0.0019	1.0196 ± 0.0019	230.4 ± 0.4	232.1 ± 0.7	−1.7 ± 0.8
	59976	2012	Whole ring	−26.6	1.0268 ± 0.0020	1.0191 ± 0.0020	230.3 ± 0.5	233.1 ± 0.4	−2.8 ± 0.6
	59977	2011	Whole ring	−26.9	1.0373 ± 0.0018	1.0296 ± 0.0018	232.7 ± 0.4	234.8 ± 0.5	−2.1 ± 0.6
	59978	2010	Whole ring	−27.1	1.0440 ± 0.0021	1.0365 ± 0.0021	234.2 ± 0.5	235.0 ± 0.6	−0.8 ± 0.8
	59984	2009	Whole ring	−27.0	1.0528 ± 0.0020	1.0453 ± 0.0020	236.2 ± 0.4	236.6 ± 0.5	−0.4 ± 0.6
5. Yamakiya (37°36.139′N, 140°40.613′E, 38 km NW of FDNPP)									
	59985	2013	Whole ring	−24.0	1.0242 ± 0.0020	1.0164 ± 0.0020	229.7 ± 0.5	232.1 ± 0.7	−2.4 ± 0.8
	59986	2012	Whole ring	−23.2	1.0313 ± 0.0021	1.0236 ± 0.0021	231.3 ± 0.5	233.1 ± 0.4	−1.8 ± 0.6
	59987	2011	Whole ring	−22.0	1.0382 ± 0.0021	1.0306 ± 0.0021	232.9 ± 0.5	234.8 ± 0.5	−1.9 ± 0.7
	59988	2010	Whole ring	−23.2	1.0476 ± 0.0018	1.0401 ± 0.0018	235.0 ± 0.4	235.0 ± 0.6	0.0 ± 0.7
	59994	2009	Whole ring	−23.1	1.0544 ± 0.0021	1.0469 ± 0.0021	236.6 ± 0.5	236.6 ± 0.5	0.0 ± 0.7
6. Namie (37°29.849′N, 141°1.484′E, 8.5 km N of FDNPP)									
	59955	2013	Whole ring	−24.2	1.0175 ± 0.0020	1.0098 ± 0.0020	228.2 ± 0.5	232.1 ± 0.7	−3.9 ± 0.8
	59956	2012	Whole ring	−24.5	1.0264 ± 0.0019	1.0187 ± 0.0019	230.2 ± 0.4	233.2 ± 0.4	−3.0 ± 0.6
	59957	2011	Whole ring	−24.0	1.0283 ± 0.0019	1.0207 ± 0.0019	230.7 ± 0.4	234.8 ± 0.5	−4.1 ± 0.6
	59958	2010	Whole ring	−24.2	1.0463 ± 0.0018	1.0387 ± 0.0018	234.7 ± 0.4	235.0 ± 0.6	−0.3 ± 0.7
	59964	2009	Whole ring	−24.2	1.0761 ± 0.0021	1.0684 ± 0.0021	241.5 ± 0.5	236.6 ± 0.5	4.9 ± 0.7

^*^The quoted uncertainties are at 1σ.

^**^Data of 2010–2012 from Levin *et al*.^11^ from which data of 2013–2015 were extrapolated by best fitting.
