# Supplementary material for: Radiocarbon Releases from the 2011 Fukushima Nuclear Accident
Source: Sci Rep. 2016 Nov 14;6:36947. doi: 10.1038/srep36947 (PMC5107918; doi:10.1038/srep36947)
Supplement: Supplementary Information [file srep36947-s1.pdf]

Radiocarbon Releases from the 2011 Fukushima Nuclear Accident

Sheng Xu[1,3,*], Gordon T. Cook[1], Alan J. Cresswell[1,3], Elaine Dunbar[1],

Stewart P.H.T. Freeman[1], Xiaolin Hou[2], Piotr Jacobsson[1], Helen R. Kinch[1],

Philip Naysmith[1], David C.W. Sanderson[1] & Brian G. Tripney[1]

[1]Scottish Universities Environmental Research Centre,

East Kilbride, G75 0QF, UK

[2]Center for Nuclear Technologies,

Technical University of Denmark,

4000 Roskilde, Denmark

[3]Institute of Environmental Radioactivity,

Fukushima University, Fukushima 960-1296, Japan

*Corresponding author: sheng.xu@glasgow.ac.uk

Supplementary Table and Figures

Table S1. Main wind directions near the FDNPP during the 2011 nuclear accident

Station	Date	Time	Main wind directions
Namie (37º29.5'N, 140º57.9'E, 47m.a.s.l, 10km NW of FDNPP)[a]			
	11th March	14:00-15:50	25% SE, 25% ESE, 17% W
Iitate (37º39.9'N, 140º43.6'E, 460m.a.s.l, 38km NW of FDNPP)[a]			
	11th March	08:00-18:00	36% W, 30% WSW, 16% SW
	12th March	08:00-18:00	46% W, 21% WNW, 10% NNW
	13th March	08:00-18:00	48% W, 23% WSW, 17% WNW
	14th March	08:00-18:00	41% WSW, 30% W, 8% WNW
	15th March	08:00-18:00	47% E, 37% ESE, 7% SE
	16th March	08:00-18:00	66% WNW, 18% W, 5% NW
Kawauchi (37º20.2'N, 140º48.5'E, 410m.a.s.l, 20km SW of FDNPP)[a]			
	21st March	08:00-18:00	25% NW, 13% NNE, 10% SE
	22nd March	08:00-18:00	42% SE, 11% W, 9% ESE
	23rd March	08:00-18:00	48% NW, 23% WNW, 20% NNW
	24th March	08:00-18:00	30% SE, 15% WSW, 10% WNW
	25th March	08:00-18:00	61% SE, 15% NNW, 7% S

[a]m.a.s.l. denotes metres above sea level.

NOAA HYSPLIT MODEL
Forward trajectories starting at 0000 UTC 01 Jan 11
GHDA Meteorological Data

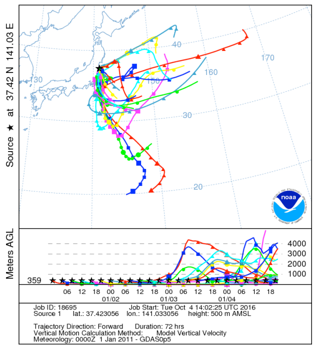

NOAA HYSPLIT MODEL
Forward trajectories starting at 0000 UTC 04 Jan 11
GHDA Meteorological Data

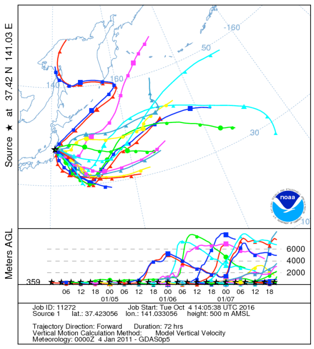

NOAA HYSPLIT MODEL
Forward trajectories starting at 0000 UTC 07 Jan 11
GHDA Meteorological Data

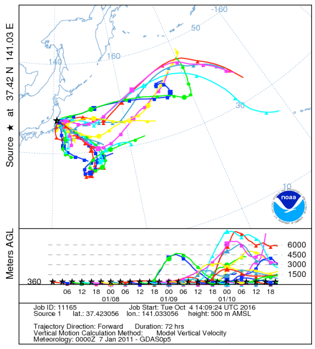

NOAA HYSPLIT MODEL
Forward trajectories starting at 0000 UTC 10 Jan 11
GHDA Meteorological Data

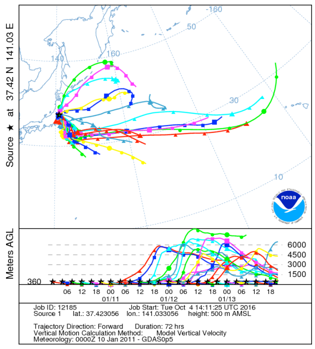

NOAA HYSPLIT MODEL
Forward trajectories starting at 0000 UTC 13 Jan 11
GHDA Meteorological Data

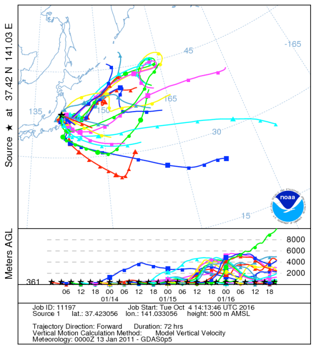

NOAA HYSPLIT MODEL
Forward trajectories starting at 0000 UTC 16 Jan 11
GHDA Meteorological Data

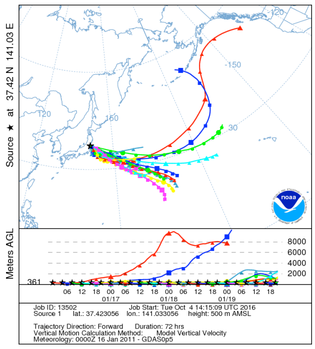

NOAA HYSPLIT MODEL
Forward trajectories starting at 0000 UTC 19 Jan 11
GHDA Meteorological Data

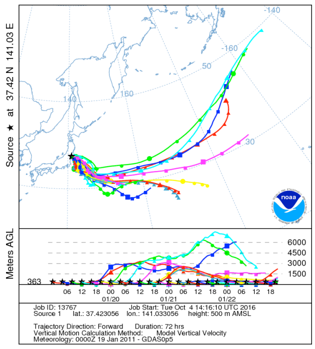

NOAA HYSPLIT MODEL
Forward trajectories starting at 0000 UTC 22 Jan 11
GHDA Meteorological Data

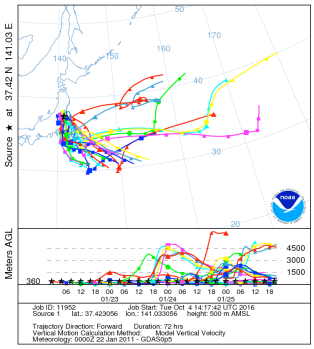

NOAA HYSPLIT MODEL
Forward trajectories starting at 0000 UTC 25 Jan 11
GHDA Meteorological Data

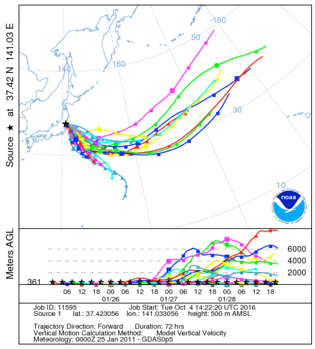

NOAA HYSPLIT MODEL
Forward trajectories starting at 0000 UTC 28 Jan 11
GHDA Meteorological Data

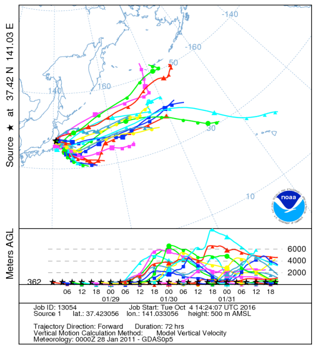

NOAA HYSPLIT MODEL
Forward trajectories starting at 0000 UTC 31 Jan 11
GHDA Meteorological Data

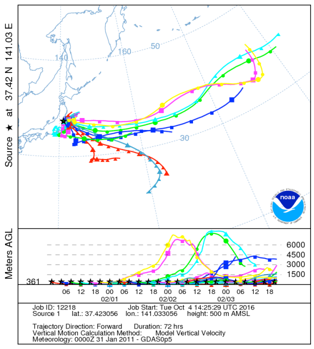

NOAA HYSPLIT MODEL
Forward trajectories starting at 0000 UTC 03 Feb 11
GHDA Meteorological Data

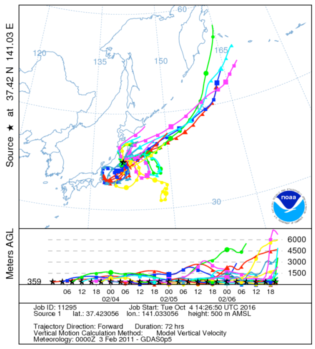

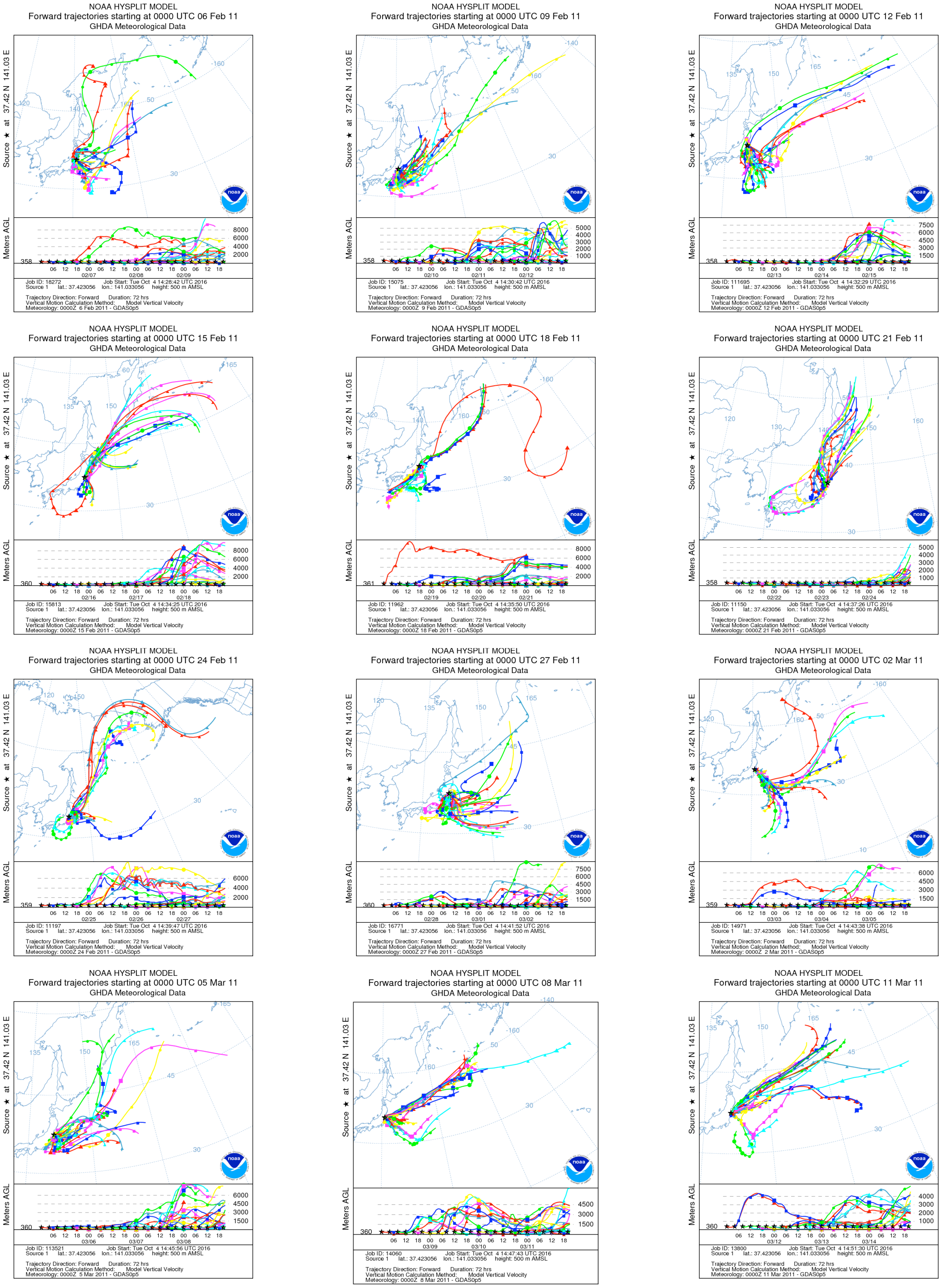

Fig. S1. Air mass forward trajectories from FDNPP during period from 1st January to 11th March 2011. A 72 h (3 day) air mass forward trajectory analysis for starting altitudes of 0 m above ground level (AGL) calculated from the FNL database of the National Ocean and Atmospheric Administration (NOAA) and simulated by using the Hybrid Single-Particle Langrangian Integrated Trajectory (HY-SPLIT) model[1,2]. References: 1. Stein, A.F., Draxler, R.R, Rolph, G.D., Stunder, B.J.B., Cohen, M.D., and Ngan, F., (2015). NOAA's HYSPLIT atmospheric transport and dispersion modeling system, Bull. Amer. Meteor. Soc., 96, 2059-2077, http://dx.doi.org/10.1175/BAMS-D-14-00110.1. 2. Rolph, G.D. (2016). Real-time Environmental Applications and Display sYstem (READY) Website (http://www.ready.noaa.gov). NOAA Air Resources Laboratory, College Park, MD)

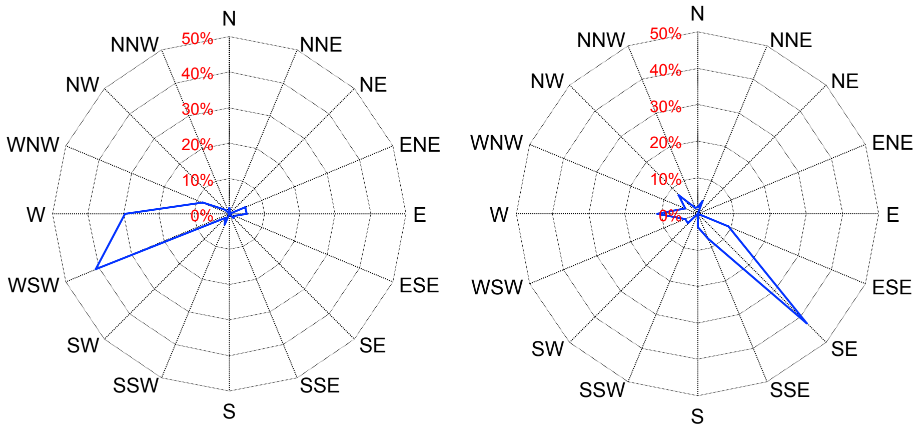

Fig. S2. Representative rose diagrams of the 10-minute-averaged wind directions between 8:00 and 18:00 on the 14th March at Iitate (left) and the 22nd March 2011 at Kawauchi (right). Data source: Japan Meteorological Agency (http://www.data.jma.go.jp)
